# Transcriptome of Porcine PBMCs over Two Generations Reveals Key Genes and Pathways Associated with Variable Antibody Responses post PRRSV Vaccination

**DOI:** 10.1038/s41598-018-20701-w

**Published:** 2018-02-06

**Authors:** Ting Yang, Fengxia Zhang, Liwei Zhai, Weiyong He, Zhen Tan, Yangyang Sun, Yuan Wang, Lei Liu, Chao Ning, Weiliang Zhou, Hong Ao, Chuduan Wang, Ying Yu

**Affiliations:** 1 Key Laboratory of Animal Genetics, Breeding, and Reproduction, Ministry of Agriculture & National Engineering Laboratory for Animal Breeding, College of Animal Sciences and Technology, China Agricultural University, Beijing, 100193 China; 2 Key Laboratory of Animal Epidemiology and Zoonosis, Ministry of Agriculture, College of Veterinary Medicine, China Agricultural University, Beijing, 100193 China; 3 Tianjin Ninghe Primary Pig Breeding Farm, Ninghe, 301500 Tianjin, China; 4 State Key Laboratory for Animal Nutrition, Key Laboratory for Domestic Animal Genetic Resources and Breeding of the Ministry of Agriculture of China, Institute of Animal Science, Chinese Academy of Agricultural Sciences, Beijing, 100193 China

## Abstract

Porcine reproductive and respiratory syndrome virus (PRRSV) is a virus susceptible to antibody dependent enhancement, causing reproductive failures in sows and preweaning mortality of piglets. Modified-live virus (MLV) vaccines are used to control PRRS in swine herds. However, immunized sows and piglets often generate variable antibody levels. This study aimed to detect significant genes and pathways involved in antibody responsiveness of pregnant sows and their offspring post-PRRSV vaccination. RNA sequencing was conducted on peripheral blood-mononuclear cells (PBMCs), which were isolated from pregnant sows and their piglets with high (HA), median (MA), and low (LA) PRRS antibody levels following vaccination. 401 differentially expressed genes (DEGs) were identified in three comparisons (HA *versus* MA, HA *versus* LA, and MA *versus* LA) of sow PBMCs. Two novel pathways (complement and coagulation cascade pathway; and epithelial cell signaling in *H. pylori* infection pathway) revealed by DEGs in HA *versus* LA and MA *versus* LA were involved in chemotactic and proinflammatory responses. *TNF-α*, *CCL4*, and *NFKBIA* genes displayed the same expression trends in subsequent generation post-PRRS-MLV vaccination. Findings of the study suggest that two pathways and *TNF-α*, *CCL4*, and *NFKBIA* could be considered as key pathways and potential candidate genes for PRRSV vaccine responsiveness, respectively.

## Introduction

The use of vaccines for controlling and eliminating virus-induced diseases is an important and complex process that highly depends on both antigen properties and host responses. Porcine reproductive and respiratory syndrome (PRRS), which is caused by the PRRS virus (PRRSV), is characterized by acute reproductive failure in sows and respiratory disorders in piglets, resulting in significant economic losses to the swine industry worldwide^1–3^. Thus, PRRS control strategies via vaccine prevention are commonly used to reduce porcine production losses^4^.

The commercially modified live-attenuated PRRSV vaccine (PRRS-MLV) is widely used to induce protective immunity in pigs. However, host immune responses against PRRSV infection induced by PRRSV vaccine cannot be maintained in the long term^5,6^. Thus, to solve this problem, sows, including gestating sows, are vaccinated several times per year with PRRS-MLV in some pig farms. PRRSV is also an antibody-dependent enhancement (ADE) virus, which may induce the susceptibility of pigs to PRRSV infection with decreasing levels of PRRSV-specific antibodies of maternal origin or with antibodies induced by exposure to PRRSV vaccination^7,8^. Numerous studies have also described that vaccination with PRRS-MLV vaccine during sow gestation may exhibit negative effects on reproductive performance, such as reduced pigs born alive and increased pigs born dead^9^. Therefore, the interaction basis and genetic fundamentals of immunogenetics between sow immune responses and PRRSV vaccines remain to be elucidated. Identification of affected genes and pathways involved in immune responses allows researchers to understand variable antibody responses of PRRSV vaccination in sows.

**Figure 1:**
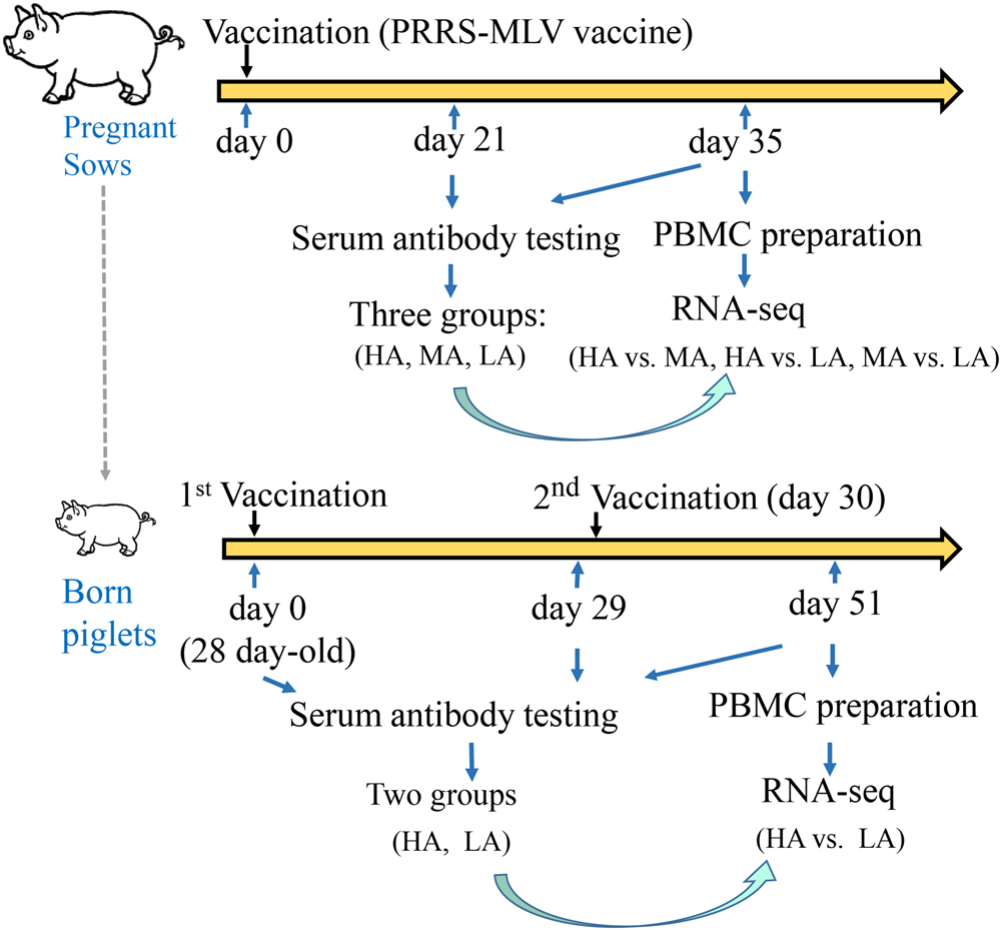
Experimental design. Pregnant sows were vaccinated with PRRS-MLV vaccine (day 0). At 21 and 35 days after vaccination, blood samples were collected. Antibody levels in sample serum at days 21 and 35 were determined using enzyme-linked immunosorbent assay (ELISA). PBMCs were isolated from blood samples at 35 days post-vaccination and used for DEG identification. Piglets were initially vaccinated with PRRS-MLV vaccine at 28 days old. Second vaccination of PRRS-MLV was performed at 58 days old. Heparinized blood samples of piglets were collected directly at 21 days after the second vaccination (79 days old). Antibody levels of piglets were determined using ELISA. PBMCs were isolated from blood samples and used for DEG identification. HA: High antibody level; MA: Median antibody level; LA: Low antibody level.

The transcriptome of peripheral blood mononuclear cells (PBMCs) not only indicates primary immune response of leukocytes but also shows the extent and dynamics of differentially expressed genes (DEGs) induced by antigens in these cells^10–12^. We first conducted genome-wide transcriptional analyses of PBMCs, which were isolated from gestating sows with high (HA), median (MA), and low (LA) levels of PRRS-antibody post-vaccination of highly pathogenic PRRSV vaccine strains TJM, to investigate the key pathways and genes involved in immune responses to PRRSV vaccination of sows. Then, transcriptomes of offspring after PRRSV vaccination were analyzed to compare common genes and immune-related pathways between two generations of pigs. Figure 1 shows our experimental design. Finally, key genes and pathways involved in porcine PBMCs post-vaccination of PRRSV-MLV were identified.

## Results

### Different immune responses of pregnant sows to PRRSV vaccination

Serum samples were isolated from the jugular vein of sows at days 21 and 35 post-vaccination of PRRSV-MLV (21 and 35 dpi) and were screened by enzyme-linked immunosorbent assay (ELISA) to evaluate antibody responses of pregnant sows induced by PRRSV vaccine.

**Figure 2:**
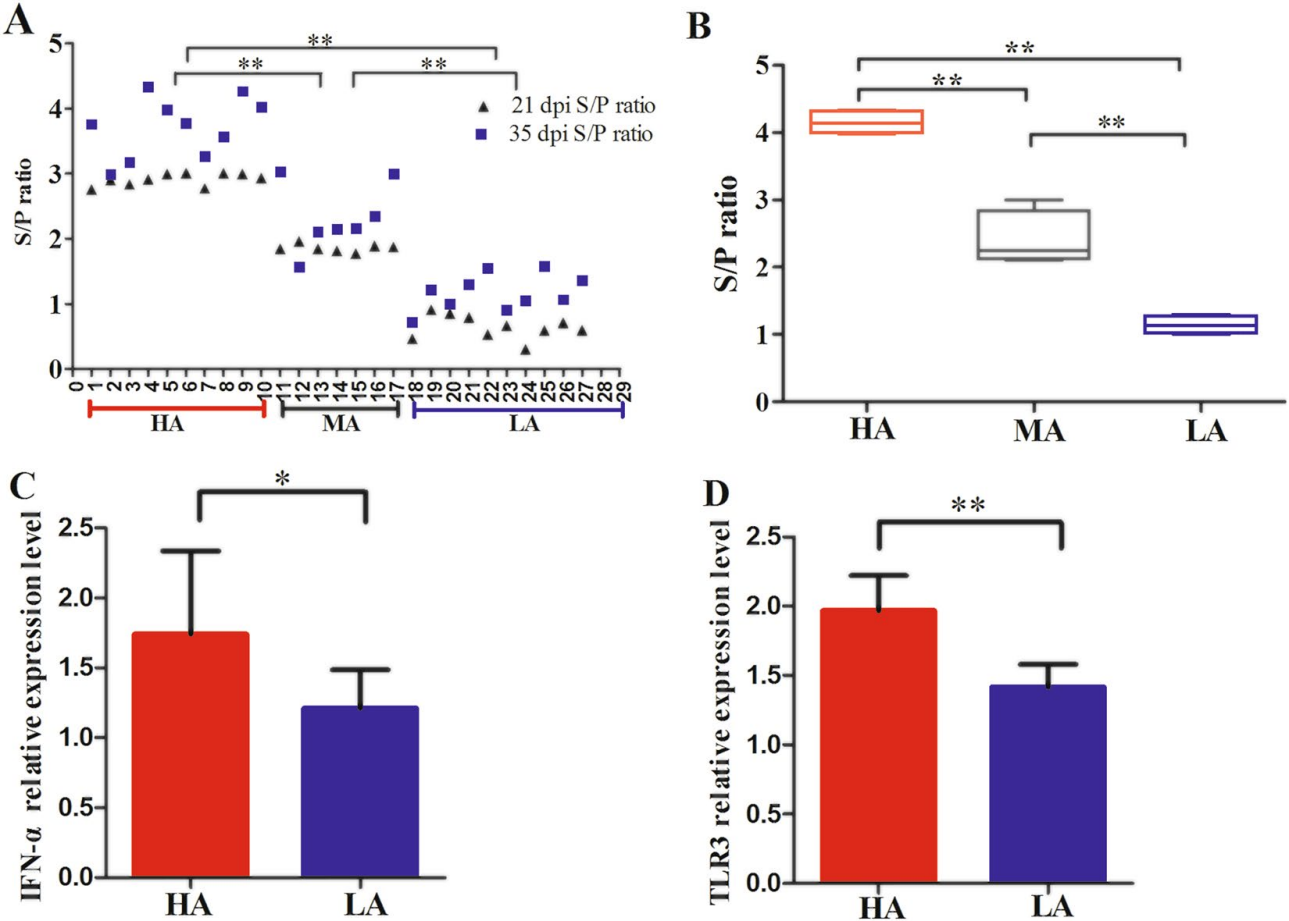
Immune responses of pregnant sows to PRRSV-MLV vaccination. (**A**) Sample-to-positive (S/P) ratio varied among the three groups at days 21 and 35 post-PRRSV-MLV vaccination (21 and 35 dpi). HA: high antibody group (*n* = 10); MA: median antibody group (*n* = 7); LA: low antibody group (*n* = 10). (**B**) S/P ratio of four individuals in each group which were selected for RNA-seq. S/P values differed among the three groups at 35 dpi (*n* = 4). (**C**) Expression levels of *IFN-α* gene in HA and LA groups with quantitative reverse transcription-polymerase chain reaction (RT-qPCR) (*n* = 4). (**D**) Expression levels of *TLR3* in HA and LA groups with RT-qPCR (*n* = 4). * and ** mean significant levels of *P* < 0.05 and *P* < 0.01 with Student’s *t*-test, respectively.

At 21 dpi, we rated 94 pregnant sows from high to low sample-to-positive (S/P) ratio (3.004 to 0.305). Then, 27 samples were selected and divided into three groups based on S/P ratio: HA group with the highest antibody level (*n* = 10, S/P = 2.911 ± 0.094), MA group with median antibody level (*n* = 7, S/P = 1.857 ± 0.059), and LA with the lowest antibody level (*n* = 10, S/P = 0.666 ± 0.190). At 35 dpi, PRRSV-specific antibody levels of these 27 pregnant sows were measured again. We observed that the antibody levels of the three groups were consistent with those at 21 dpi. The findings were as follows: the HA group incurred high S/P ratio (*n* = 10, S/P = 3.714 ± 0.461), the MA group with median antibody level (MA, *n* = 7, S/P = 2.335 ± 0.519), and the LA group with low antibody level (LA, *n* = 10, S/P = 1.177 ± 0.277) (Fig. 2A). Four sows in each group at 35 dpi were randomly selected for RNA sequencing (RNA-seq), and their specific antibody levels differed significantly among the three groups (*P* < 0.05) (Fig. 2B). Quantitative reverse transcription polymerase chain reaction (RT-qPCR) was conducted in PBMCs to assess responses of known host-molecular signature genes (*IFN-α* and *TLR3*) to viral vaccine^13,14^ between the HA and LA groups. Results indicated significantly higher transcriptional expression levels of *IFN-α* and *TLR3* in the HA group than in the LA group (Fig. 2C,D, respectively; *P* < 0.05).

### Differentially expressed genes (DEGs) in pregnant sows with different antibody levels post-PRRSV vaccination

Subsequently, four individuals in each group were used for identifying DEGs of sow PBMCs via RNA-seq to analyze genome-wide transcriptional expression differences among the three groups. Under the criteria of |fold change| (|FC|) > 1.5 and *q* < 0.05, 401 DEGs, including 91 up-regulated and 310 down-regulated DEGs, were identified by comparing the three groups. During comparison of HA *versus* MA, 89 significantly DEGs were detected (Table 1). Among these 89 genes, 45% were up-regulated (9/20) with |FC| > 2, whereas 36% were down-regulated (25/69) with |-FC| > 2. Genes *MYL4* and *TMP-CH242-74M17.6* showed 10 times upregulation and downregulation in HA sows compared with MA sows, respectively. Table 1 lists the numbers of DEGs during comparison of HA *versus* LA and MA *versus* LA. Results revealed the highest number of DEGs in HA *versus* LA, whereas the least DEG number was detected in HA *versus* MA. *MYL4* was also 10 times up-regulated in HA compared with LA. *IL-1α*, *IL-1β*, and *ENSSSCG00000009469* displayed 10 times downregulation in HA compared with that in LA. These data indicate strong inducement of proinflammatory cytokines interleukin (IL)-1α and IL-1β in LA pigs.

**Table 1:** Number of differentially expressed genes (DEGs) disclosed in three comparisons of sows after porcine reproductive and respiratory syndrome modified live-attenuated (PRRS-MLV) vaccination.

Criteria	Expression modes	HA *vs* MA	HA *vs* LA	MA *vs* LA
*P* < 0.05, fold change	Total (401)	89	159	153
|FC| > 1.5	up (91)	20	17	54
	down (310)	69	142	99
*P* < 0.05, |FC| > 2	Total (175)	34	78	63
	up (35)	9	10	16
	down (140)	25	68	47
*P* < 0.05, |FC| > 10	Total (11)	2	4	5
	up (3)	1	1	1
	down (8)	1	3	4

**Figure 3:**
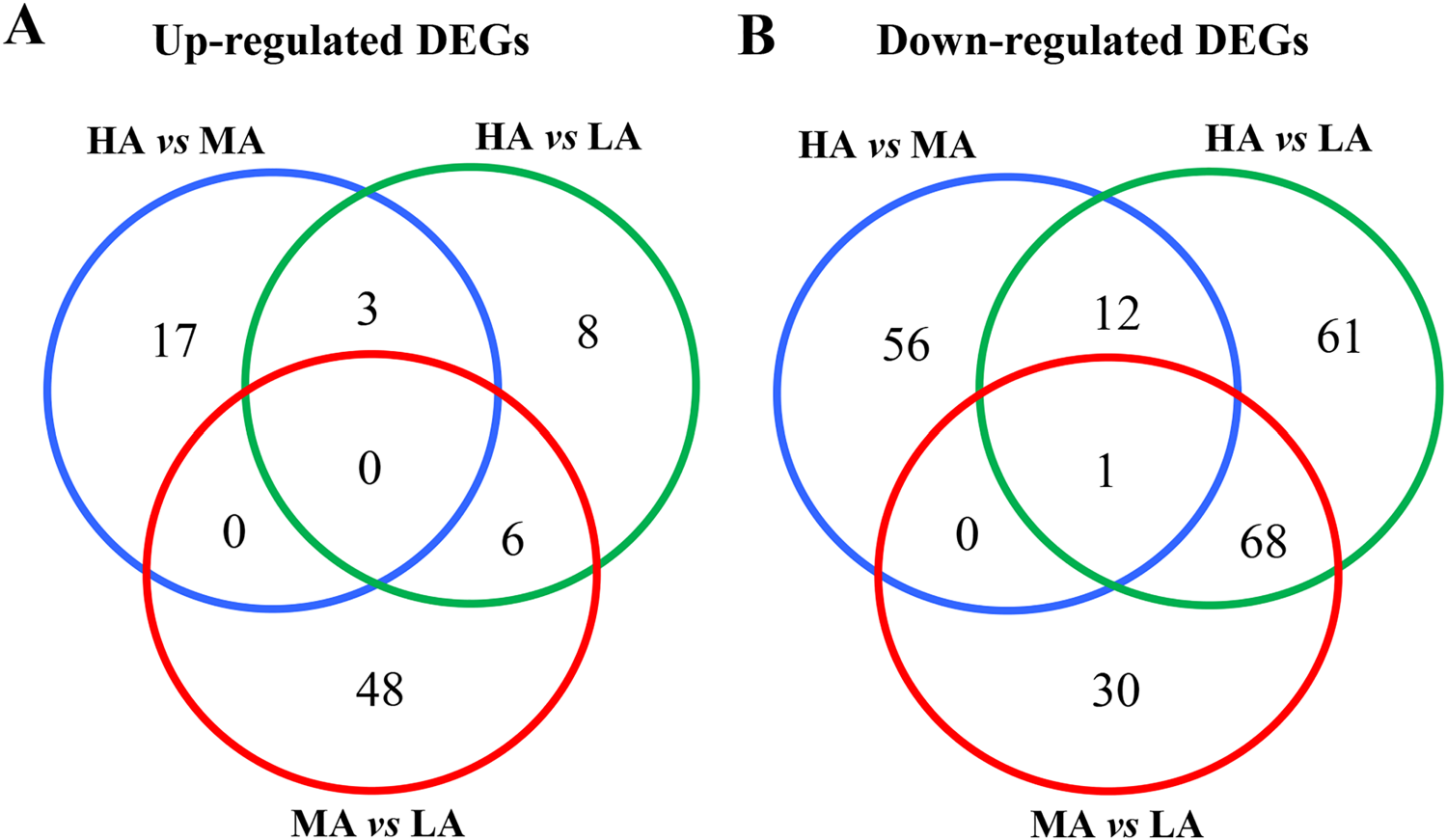
DEGs in different comparisons in sow PBMCs. (**A**,**B**) Venn diagrams of up-regulated and down-regulated DEGs after vaccination against PRRS.

Figure 3 shows the commonly up-regulated and down-regulated DEGs observed among the three comparisons. Data indicated that down-regulated DEGs between HA *versus* LA and MA *versus* LA (68 DEGs) were 11 times higher than up-regulated DEGs (6 DEGs). Only one common gene (*TNF-α*) was detected in three comparisons in down-regulated DEGs (Fig. 3B).

According to biological pathways of involved DEGs, we proposed that general immune responses of pigs are susceptible to modulation by PRRSV vaccine. Supplementary Figure S1 describes the transcriptional responses and interprets vaccine–host interaction of sow PBMCs after PRRS-MLV vaccination. As shown in the figure, we observed significant downregulation of various important inflammatory mediators (tumor necrosis factor (TNF)-α, IL-1α, and IL-1β) and chemokines (chemokine (C-C motif) ligand (CCL) 2, CCL4, CCL8, chemokine (C-X-C motif) ligand 2 (CXCL2), and CXCL8) in the HA group compared with that in the LA group. However, seven genes (*SLA-DOB*, *C-JUN*, *EGR1*, *CD69*, *MAL*, *CDH2*, and *FOS*) were regulated in opposite directions when comparing HA *versus* LA and HA *versus* MA (Supplementary Figure S1).

### Validation of RNA sequencing by RT-qPCR

Seven genes, including three up-regulated (*IFN-γ*, *CXCR4*, and *CCR1*) and four down-regulated ones (*CCL8*, *CCL4*, *IL1B*, and *CXCL8*), were selected for RT-qPCR confirmation to verify the DEGs obtained by RNA-seq. Results were presented as |FC|s of gene expression levels normalized to *GAPDH* gene. Expression profiles of genes determined by RT-qPCR were consistent with those of RNA-seq results (Supplementary Figure S2), confirming the reliability of our RNA-seq data.

### Gene ontology (GO) terms and Kyoto Encyclopedia of genes and genomes (KEGG) pathways of DEGs in sows after PRRSV vaccination

GO annotates genes to biological, cellular, and molecular terms in a hierarchically structured way, whereas KEGG divides DEGs according to functional pathways. We therefore further analyzed GO terms and KEGG pathways of 401 DEGs using Database for Annotation, Visualization and Integrated Discovery (DAVID) bioinformatics resources (version 6.7).

With regard to the 91 up-regulated DEGs, we found no GO terms after three comparisons. However, two pathways (ErbB signaling pathway and T cell receptor signaling pathway) were detected during comparison of MA *versus* LA, and related DEGs (*C-JUN*, *HBEGF*, *IFN-γ*, and *FOS*) were up-regulated in the MA group compared with LA (Table 2).

**Table 2:** Functional pathways detected in peripheral blood mononuclear cells (PBMCs) of sows.

	Pathway	DEGs	*P* value
**Up-regulated**			
MA vs. LA*	ErbB signaling pathway	*C-JUN*, *HBEGF*	0.026
	T cell receptor signaling pathway	*C-JUN*, *IFN-GAMMA*, *FOS*	0.039
**Down-regulated**			
HA vs. MA	Epithelial cell signaling in *H. pylori* infection	*C-JUN*, *HBEGF*	0.031
HA vs. LA	Cytokine-cytokine receptor interaction	*IL1A*, *IL18*, *TNFRSF8*, *OSM*, *CXCL2*, *TNFSF15*, *CCL8*, *TNFSF9*, *IL1B*, *TGFB2*, *CXCL8*, *PF4*	2.22E-6
	nucleotide-binding oligomerization domain (NOD)-like receptor signaling pathway	*NFKBIA*, *IL18*, *CCL8*, *MEFV*, *IL1B*, *CXCL2*, *CXCL8*, *NLRP3*	3.20E-6
	Toll-like receptor (TLR) signaling pathway	*NFKBIA*, *MAP3K8*, *TLR2*, *MYD88*, *IL1B*, *CD14*, *CXCL8*	6.32E-4
	Chemokine signaling pathway	*NFKBIA*, *GNG11*, *CXCL2*, *CCL8*, *NCF1*, *CXCL8*, *PF4*	7.01E-4
	Complement and coagulation cascades	*VWF*, *SERPINE1*, *C2*, *C4BPA*, *F3*	0.005
	Epithelial cell signaling in *H. pylori* infection	*NFKBIA*, *CXCL2*, *CXCL8*	0.035
MA vs. LA	NOD-like receptor signaling pathway	*NFKBIA*, *IL18*, *CCL8*, *MEFV*, *IL1B*, *CXCL2*, *CXCL8*	1.26E-5
	Cytokine-cytokine receptor interaction	*IL1A*, *IL18*, *CXCL2*, *TNFSF15*, *CCL8*, *TNFSF9*, *IL1B*, *CXCL8*, *PF4*	3.22E-4
	Chemokine signaling pathway	*NFKBIA*, *GNG11*, *CXCL2*, *CCL8*, *NCF1*, *CXCL8*, *PF4*	9.82E-4
	TLR signaling pathway	*NFKBIA*, *MAP3K8*, *TLR2*, *IL1B*, *CXCL8*	0.0016
	Complement and coagulation cascades	*VWF*, *SERPINE1*, *F3*, *CR2*	0.021
	Hematopoietic cell lineage	*IL1A*, *GP5*, *IL1B*, *CR2*	0.038

*HA: group with high antibody level; MA: group with median antibody level; LA: group with low antibody level.

**Figure 4:**
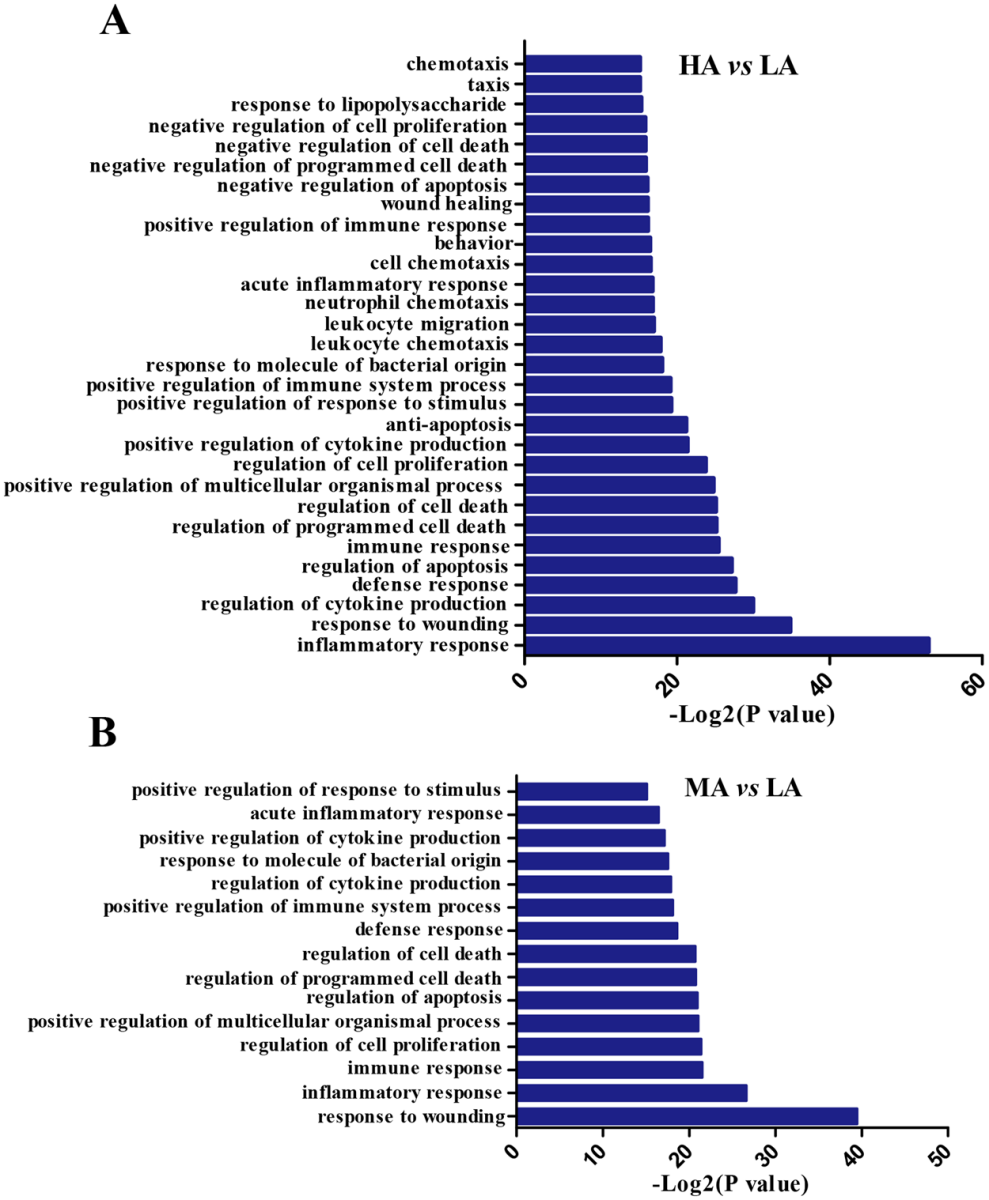
Gene Ontology (GO) term analysis of the DEGs. Biological process enriched by down-regulated DEGs in HA *versus* LA (**A**) and MA *versus* LA (**B**) induced by PRRSV-MLV vaccine in sows.

For the down-regulated DEGs, 30 enriched GO terms (Fig. 4A) and six pathways (Table 2) were obtained in comparison of HA *versus* LA. Significantly enriched biological processes were related to immune response functions, including inflammatory responses, response to wounding, cytokine production, defense response, cell proliferation, leukocyte chemotaxis and migration, stimulus response, and immune system processes (Fig. 4A). With regard to the six pathways detected in comparison of HA *versus* LA (Table 2), epithelial cell signaling in *H. pylori* infection serves as a novel pathway related to PRRSV vaccination, whereas the other five pathways were reportedly involved in immune responses to PRRSV infection^15^ and PRRSV vaccination^16^.

During comparison of MA *versus* LA, 15 enriched GO terms were obtained from down-regulated DEGs (Fig. 4B). Such biological processes involved in immune responses were similar to those in comparison of HA *versus* LA, in which six pathways were obtained (Table 2). Among these pathways, a unique hematopoietic cell lineage was observed during comparison. The remaining five pathways were consistent with those in comparison of HA *versus* LA. Results showed the identical key pathways enriched in innate and cell-mediated immune responses between comparisons of HA *versus* LA and MA *versus* LA.

### Protein–Protein interaction (PPI) of DEGs in sow and piglet PBMCs induced by PRRSV vaccination

**Figure 5:**
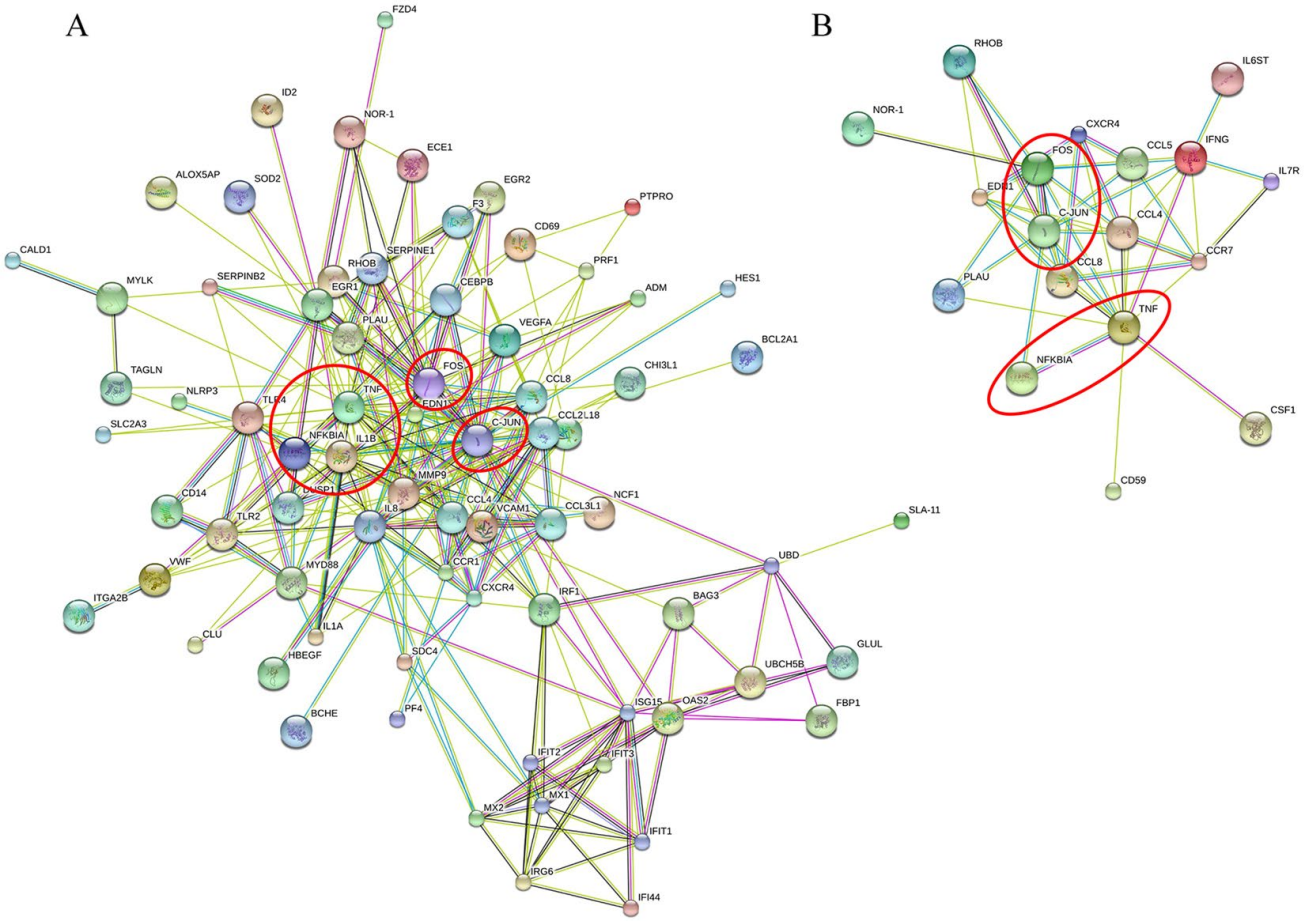
Protein–protein interaction network of DEGs produced by STRING analysis. STRING analysis was used to analyze DEGs in PBMCs of sows (**A**) and piglets (**B**). Differently colored lines represent seven types of evidence used in predicting associations. Red line: fusion evidence; blue line: co-occurrence evidence; yellow line: text mining evidence; green line: neighborhood evidence; purple line: experimental evidence; light blue line: database evidence; and black line: co-expression evidence.

PPI analysis was performed, and we observed significant interplay in a subset of 73 proteins encoded by DEGs in sows (Fig. 5A). In the network, the proteins encoded by *C-JUN*, *FOS*, *CCL2*, *CCL4*, *CCL8*, *CLU*, *CXCR4*, *ID2*, *IL1A*, *IL1B*, *IL8*, *IRG6*, *MX1*, *MX2*, *MYD88*, *NFKBIA*, *TNF-α*, *VEGFA*, *IRF1*, *PF4*, and *TLR4* were highly interconnected (Fig. 5A). Considering significant differences in the two extreme sow groups (HA and LA) for antibody levels, we followed up on the offspring of these groups and performed further PPI analysis. Results showed that the proteins encoded by *C-JUN*, *FOS*, *CCL4*, *CCL8*, *CXCR4*, *NFKBIA*, and *TNF-α* were also present as pivotal nodes in the HA and LA groups in the next generation (Fig. 5B). These data suggest that the common node genes (*C-JUN*, *FOS*, *NFKBIA*, and *TNF-α*), which are encircled in Fig. 5A,B, play the most significant roles in pregnant sows and piglets induced by PRRSV vaccine.

### Transcriptomic homogeneity in pregnant sows and their offspring

**Figure 6:**
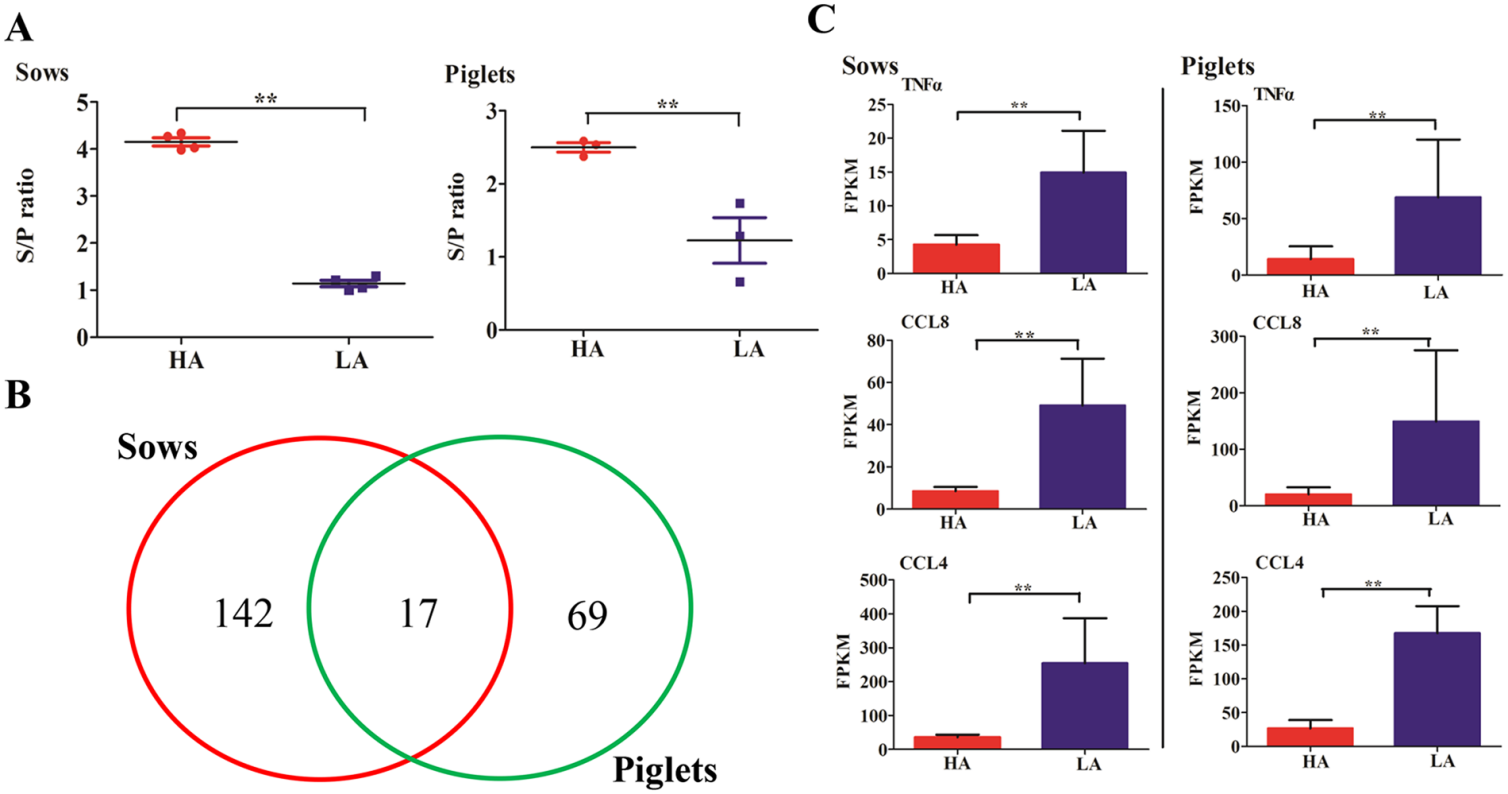
Unique and mutual transcriptomic changes between sows and offspring in comparison of HA *versus* LA. (**A**) S/P ratio between sows (*n* = 4) and piglets (*n* = 3). (**B**) Venn diagram showing the number of DEGs overlapping in sows and their offspring. (**C**) Expression trend of three common DEGs (*TNFα*, *CCL8*, and *CCL4*) between sows (*n* = 4) and piglets (*n* = 3). * and ** mean *P* < 0.05 and *P* < 0.01 with Student’s *t*-test, respectively.

Upon follow-up of piglets of pregnant sows, we compared antibody levels and DEGs in the two generation of pigs for comparison of HA *versus* LA (Fig. 6A,B). Results showed that during comparison of HA *versus* LA, 17 out of 159 DEGs (10.69%) in sows were also detected in their offspring (Fig. 6B). Signaling pathways involving toll-like receptor (TLR) signaling pathway were also affected in both sows and their offspring. Out of 17 common genes between sows and their piglets, 15 genes (88.24%) displayed the same expression tendency in comparison of HA *versus* LA. As shown in Fig. 6C, *TNF-α*, *CCL8*, and *CCL4* gene expression tendency in the HA *versus* LA groups in offspring agreed with that in sows. Supplementary Figure S3A summarizes the antibody levels of vaccinated animals compared with naive piglet serum responses. Findings indicated the lower PRRSV antibody levels in newborn pre-immune piglets (mean of their S/P ratio is 6.07E-18) in comparison with vaccinated animals. Investigation by RT-qPCR on biological variations in *TNF-α*, *CCL4*, and *CXCL8* genes in naive piglets showed increased sensitivity compared with PRRSV antibody levels (Supplementary Figure S3B). Data indicated that common, sensitive, and specific gene-expression patterns in two successive generations can provide further insights into host–vaccine interaction post-PRRSV vaccination.

**Figure 7:**
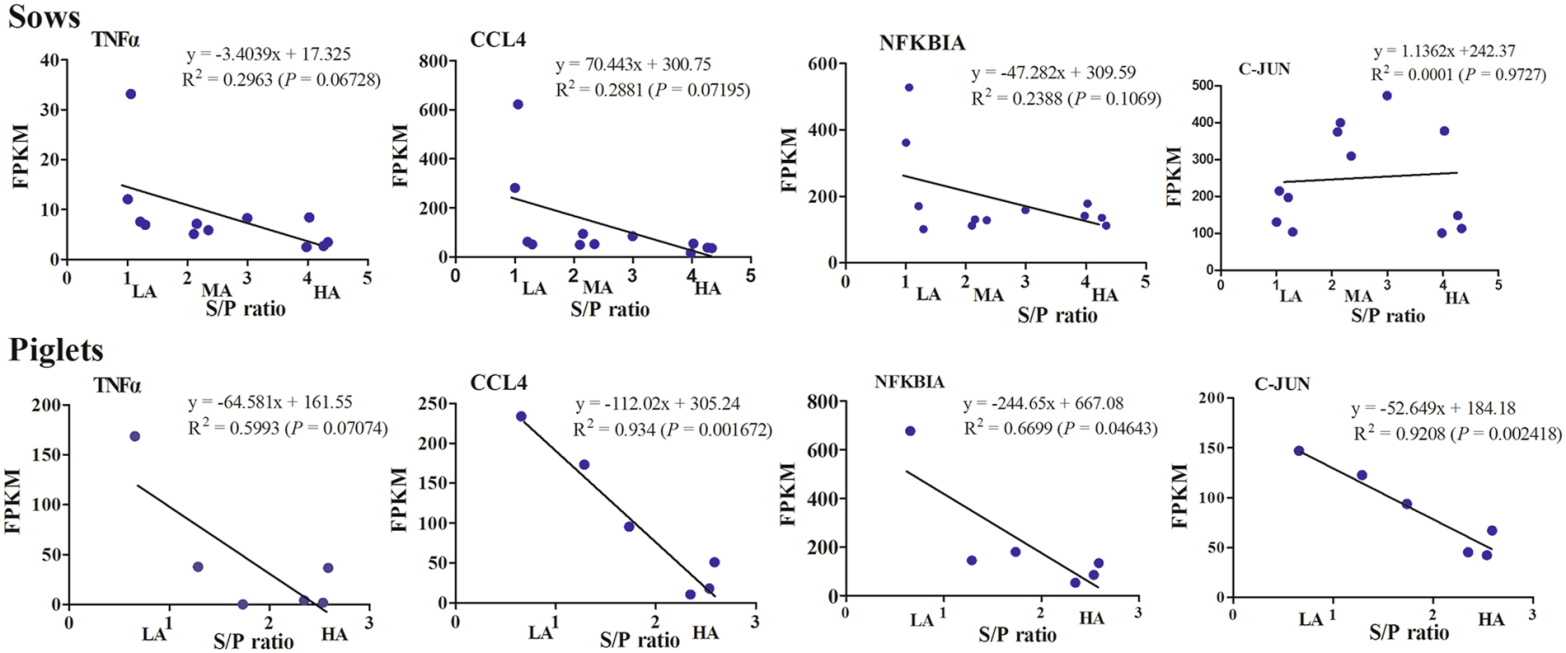
Regression analysis of divergent PRRSV-MLV-specific antibody levels (x-axis) with gene expression levels (y-axis) in sows and piglets.

Remarkably, we observed negative regressions between expression levels of *TNF-α*, *CCL4*, and *NFKBIA* with PRRS-MLV-specific antibody levels in HA, MA, and LA sows (upper panel in Fig. 7). Similar expression trends of the three genes were observed in piglets in comparison of HA *versus* LA (lower panel in Fig. 7). Thus, sows with low PRRSV antibody levels presented high transcript abundances of cellular immune-related genes.

## Discussion

PRRS is an economically important and panzootic disease leading to reproductive failure in breeding pigs and respiratory tract sickness in piglets. Simultaneously, the use of vaccines to control PRRS is a tough choice for pig farms as PRRSV vaccination poses safety risks on virulent reversion of modified live vaccines (MLV) and depends on immune responses of pigs to the vaccine^17,18^. This study investigated genome-wide transcriptional changes in PBMCs associated with high, medium, and low antibody responses over two generations of Landrace pigs after PRRSV vaccination. Finally, our results revealed several significant genes and pathways involved in variable antibody responsiveness of pregnant sows and their offspring post-PRRSV vaccination via next-generation sequencing technology.

Our results demonstrated that more than 400 DEGs and 12 KEGG pathways were involved in gestating sows of HA, MA, and LA PRRSV-antibody levels. A total of 17 DEGs and two pathways recurred in piglets during comparison of HA *versus* LA. To integrate DEGs in sows with healthy and PRRSV susceptibility-related quantitative trait loci (QTLs), we matched positions of DEGs to QTL regions for health traits (including PRRSV susceptibility) in the pig QTL database (http://www.animalgenome.org/cgi-bin/QTLdb/SS/index). Our search discovered that 19 DEGs were distributed in 17 different QTL regions associated with pig healthy and PRRSV susceptibility-related traits (Table 3). Out of 19 DEGs, *MEGF9*, *NDRG1*, *SLA-8*, and *C2* were located within QTL regions, which were validated to display remarkable genetic effects on PRRSV susceptibility^19^, whereas *STX11* was located in the QTL region of CD4-positive leukocyte percentage. Thus, these five genes were suggested to be powerful candidate genes for PRRSV vaccine responsiveness in porcine. Transcriptional responses and underlying mechanisms associated with high, medium, and low level of neutralizing antibody must be measured to improve the protection of PRRSV vaccination.

**Table 3:** Information on overlapping between DEGs and quantitative trait loci (QTLs) related to healthy traits in sows.

Gene symbol	Gene Position	QTL region	Gene ID	Healthy traits of QTL effect
*STX11*	Chr1:23480022-23510485	Chr1:20064508-24647055	ENSSSCG00000004125	CD4-positive leukocyte percentage
*MEGF9*	Chr1:292733749-292827889	Chr1:292036476-292998740	ENSSSCG00000005506	PRRSV susceptibility
*NDRG1*	Chr4:7894534-7931354	Chr4:7002305-7975375	ENSSSCG00000005944	PRRSV susceptibility
*SLA-8*	Chr7:27209603-27630394	Chr7:27019705-27999311	ENSSSCG00000001396	PRRSV susceptibility
*C2*	Chr7:27778160-27819252	Chr7:27019705-27999311	ENSSSCG00000001422	PRRSV susceptibility
*TLR4*	Chr1:289775822-289785847	Chr1:289784892-289784932	ENSSSCG00000005503	Salmonella shedding status
*MRVI1*	Chr2:52130620-52274846	Chr2:52143832-52143872	ENSSSCG00000020720	Mean corpuscular hemoglobin content, Mean corpuscular volum
*GCNT4*	Chr2:85693064-85722431	Chr2:85683635-85821787	ENSSSCG00000029710	Cholesterol level
*TNFRSF8*	Chr6:66086486-66122079	Chr6:61924724-66465212	ENSSSCG00000003437	C3c concentration, Haptoglobin concentration
*SLA-11*	Chr7:26674362-26716913	Chr7:26674137-26796806	ENSSSCG00000001341	Mean corpuscular hemoglobin content
*EDN1*	Chr7:9152653-9159251	Chr7:7974684-11402680	ENSSSCG00000001050	Phosphate level
*GMPR*	Chr7:12850723-12893472	Chr7:12837931-12928726	ENSSSCG00000001064	Mycoplasma hyopneumoniae antibody titer
*PTPN13*	Chr8:141369845-141583918	Chr8:139007531-141371437	ENSSSCG00000026655	Plateletcrit
*MYL4*	Chr12:16799291-16813119	Chr12:16800333-16800373	ENSSSCG00000017307	hematocrit
*MAPT*	Chr12:17123471-17172747	Chr12:17124741-17124781	ENSSSCG00000017311	hematocrit
*CDC42E*	Chr12:7918389-7939265	Chr12:4823589-8879317	ENSSSCG00000017245	Hemoglobin
*HES1*	Chr13:140633173-140635748	Chr13:140120987-141398868	ENSSSCG00000026362	Triglyceride level
*GP5*	Chr13:140881372-140883295	Chr13:140120987-141398868	ENSSSCG00000031035	Triglyceride level
*IGF2BP2*	Chr13:133022540-133065865	Chr13:133062856-133622872	ENSSSCG00000011795	HDL/LDL ratio

In the current study, two immune response-related pathways (i.e., TLR signaling pathway and T cell receptor signaling pathway) were exposed to PRRSV vaccination responsiveness in the two generations of pig. Especially, TLR signaling pathway plays a significant role in adaptive immunity^16,20^. This pathway is primarily represented by *CD14*^21^, which was significantly up-regulated in LA pigs and moderately up-regulated in MA pigs compared with that in the HA group after PRRSV-MLV vaccination (Table 2 and Supplementary Figure S1). On the other hand, in PPI analysis, CD14 was closely interconnected with TLR2 and TLR4 (Fig. 5), which are members of the TLR family and may play roles in virus recognition^22,23^. T cell receptor pathway performs a central role in regulation of thymocyte development and T cell differentiation^24^. The present study observed that DEGs (*C-JUN*, *IFN-γ*, and *FOS)* in the pathway were up-regulated in MA pigs compared with that in LA animals. TLR signaling pathway and T cell receptor pathway are known to be involved in host–vaccine interaction^14,22^; however, further research at more time points is needed to dissect whether these genes in the two pathways actually affect the responsiveness of PRRSV vaccination.

The present study reported for the first time the association of two pathways (i.e., complement and coagulation cascade pathway and epithelial cell signaling in *H. pylori* infection pathway) with PRRSV vaccination responsiveness. The complement and coagulation cascade pathway exhibits a first line of defense against pathogens and other invaders that may enter circulation and affect inflammatory responses^25^. In this study, epithelial cell signaling in *H. pylori* infection known as infectious disease-related pathway was detected for the first time to be associated with PRRSV vaccination, indicating that animal immune responses to pathogens can share certain evolutionary conservation^26^. DEGs (*NFKBIA* and *CXCL8*) in the two pathways showed the potential to distinguish antibody levels in PRRSV-vaccinated pigs; these DEGs were down-regulated in HA pigs compared with that in the LA group.

Cytokines and chemokines in cellular immune response play a pivotal role in the initial stage of post-vaccination responsiveness^27,28^. We observed that cytokine *TNF-α* and chemokine *CCL4* were down-regulated in pigs with high antibody levels. Similar downregulation of *CCL4* was reported in piglets with high humoral immune responses (ELISA) after primary PRRSV vaccination^29^. These negative regressions (Fig. 7) were validated by adding more sows (Supplementary Figure S4). TNF-α is a primary mediator of systemic inflammation and is one of the cytokines participating in acute phase reaction^30^. TNF-α plays a significant role in efficient innate immunity, antiviral effect initiation, activation, maturation and differentiation of monocyte/macrophage lineages, and stimulation of natural killer cells to produce *IFN-γ*^31,32^. Chemokine CCL4 (MIP-1β) is the most potent chemoattractant and mediator of virus-induced inflammation *in vivo*^33^. These findings imply that cellular immune response could be improved in sows and piglets with low antibody levels after PRRSV vaccination to compensate for inferior humoral immune responses.

Encoded proteins of *FOS*, *C-JUN*, and *NFKBIA* were identified as central nodes of vaccine-induced transcriptional network in PBMCs of PPI networks (Fig. 5A). The proto-oncogenes *FOS* and *C-JUN* work mutually as derivable transcription factors in signal transduction. FOS is a nuclear phosphoprotein involved in important cellular events, including cell proliferation, differentiation, and survival, and controlled replication of hepatitis C virus^34,35^. C-JUN is known as a regulator of primary biological processes^35,36^ and downregulates p53, which is a suppressor of apoptosis and cell differentiation^37,38^ and encodes an avian sarcoma virus-like protein^39^. We observed that expression trends of key node genes fluctuated between the two generations (Fig. 7). Therefore, further study is warranted to dissect whether the variably expressed genes are involved in variable PRRSV vaccination responsiveness in pigs.

The third node gene *NFKBIA* (I*κ*-Bα) encodes NFKB inhibitor alpha (NFKBIA) protein, which is a repressor of nuclear factor (NF)-*κ*B transcription by covering nuclear localization signals of NF-*κ*B proteins and maintaining their inactivity in cytoplasm^40^. NFKBIA also inhibits NF-*κ*B from binding to DNA; thus, NF-*κ*B cannot function as a transcription factor^41,42^. NF-*κ*B is a universal transcription factor that induces several relevant downstream signal transductions, resulting in upregulation of proinflammatory cytokines, type-I interferon, and chemokines, which promote inflammatory processes, apoptosis, and phagocytosis^40,43^. Our results suggest that NFKBIA is the node with the most potential to prevent inflammatory process following PRRS-MLV vaccination in PBMCs of sows (Fig. 5A). With *NFKBIA* showing a similar trend in the next generation of sows post-PRRS-MLV vaccination (Fig. 7, Supplementary Figure S4), these data suggest that *NFKBIA* could be a candidate gene relevant with pig PRRSV vaccine responsiveness.

In conclusion, we performed genome-wide transcriptional profiles to investigate genes, pathways, and networks related with immune responses in PBMCs of Landrace sows and their offspring following PRRS-MLV vaccination. This study revealed 401 DEGs in three comparisons (HA *versus* MA, HA *versus* LA, and Ma *versus* LA) and identified *NFKBIA*, *TNF-α*, *CXCL8*, and *CCL4* as the most promising candidate genes that can contribute to the interaction between sows and PRRS-MLV vaccine. *TNF-α*, *CCL4*, and *NFKBIA* are more possibly related with variable antibody responses in PRRSV vaccination in both sows and offspring. On the other hand, two pathways were detected to be associated with sow PRRSV vaccine responsiveness: complement and coagulation cascade pathway and epithelial cell signaling in *H. pylori* infection pathway. These DEGs and novel pathways provide new insights into the molecular mechanism of immune response in sow and piglet PRRS vaccination. Future studies will be needed to identify these novel gene functions in PRRSV vaccine responsiveness.

## Materials and Methods

### Animals, vaccination, and PRRS antibody detection

All the following procedures involving animals were approved by the Animal Welfare Committee of China Agricultural University in Beijing, China. Animal experiments were also conducted in strict accordance with regulations and guidelines established by this committee (permit number: DK996).

#### Sows

Landrace pregnant sows (1–3 months of pregnancy) were obtained from Tianjin Ninghe Primary Pig Breeding Farm (Tianjin, China). Sows were reared in the same normal conditions with natural room temperature and light, fed twice daily, and provided ad libitum access to water. Pedigree information was available for all animals. In this study, sows were free of serum antibody of PRRSV, classical swine fever virus, and pseudorabies virus, as confirmed by ELISA detection.

Figure 1 outlines the experimental design. A total of 94 Landrace pregnant sows in the middle stages of gestation (30–80 days of pregnancy, 2nd–3rd parity, and age of approximately 2.5 years old) were inoculated (day 0) intramuscularly with one dose (1 mL) of PRRS-MLV and named highly pathogenic porcine PRRS vaccine (live strain TJM-F92) (Pfizer, USA). Sow blood samples were collected for analysis at days 21 and 35 post-inoculation of PRRS-MLV vaccine. PBMCs at day 35 were isolated from heparinized blood collected by Ficoll–Hypaque (TBD, Tianjin, China) gradient centrifugation and stored at −80 °C. Sera at two time points were isolated from blood samples by centrifuging at 2000 *g* for 10 min at room temperature.

Serum PRRSV antibody levels were determined using an ELISA kit (HerdCheck PRRS 3X, IDEXX Laboratories Inc., USA) according to manufacturer’s protocol. Optical density (OD) of each well was determined at 630 nm using a microplate reader (Multiskan MK3, Thermo Scientific, USA). Presence or absence of PRRSV-specific antibodies in samples was determined by calculating S/P ratio. S/P ratio was calculated according to the following equation: S/P ratio (%) = 100 × [(OD of test sample—mean OD of negative controls)/(mean OD of positive controls—mean OD of negative controls)]. Samples were considered positive for PRRSV antibody when S/P ratio reached 0.4 or higher^44^. Three groups (HA, MA, and LA) with different PRRSV antibody levels were classified according to S/P ratio at 21 dpi and confirmed by S/P ratio at 35 dpi. Four sows in each group (HA, MA, and LA) were randomly selected to perform RNA-seq according to S/P ratio at 35 dpi.

#### Piglets

Sow piglets in the same farm were earmarked by small cuts with a sharp pincer to record PRRSV antibody levels of their offspring piglets from the HA and LA groups. All piglets were raised under the same standard indoor conditions according to their growth stages. In detail, the piglets were raised in farrowing house from birth to 28 days old. Then, the piglets were moved to nursery pens until their weight reached approximately 30 kg. Subsequently, the animals were moved to growing–finishing pens to be sold out as breeders (~150 days old). According to routine vaccination program of the farm, the piglets were initially vaccinated (28 days old) intramuscularly with one dose (1 mL) of PRRS-MLV vaccine. The second vaccination was administered after 30 days (58 days old). Heparinized blood samples were collected directly 21 days after the second vaccination (79 days old). Levels of serum PRRSV antibody at 79 days old were measured by an ELISA kit (HerdCheck PRRS 3X, IDEXX Laboratories Inc., USA). Finally, three animals from each group were selected as follows: HA piglet group from the HA sow group (S/P = 2.499 ± 0.111) and LA piglet group from the LA sow group (S/P = 1.227 ± 0.542). The selected animals were used to conduct transcriptome examination with PBMCs at 79 days old. Newborn piglets (*n* = 4) in a naive population were not vaccinated but served as controls.

### RNA isolation, cDNA library preparation, and RNA-seq

Total RNA of pig PBMCs was extracted directly from each sample using TRIzol (Invitrogen Life Technologies, Carlsbad, CA, USA) according to manufacturer’s protocol. RNA quality and integrity were measured with the Agilent Bioanalyzer 2100 (Agilent Technologies, Santa Clara, CA, USA). All RNA integrity numbers of samples were above nine. The 28S:18S rRNA ratios of all samples ranged from 1.7 to 2.4.

Equivalent amount (4 µg) of total RNA from PBMCs of each pig was used to construct RNA-seq libraries. Poly-A-containing mRNA was purified from total RNA using Oligo (dT) magnetic beads (NEB, Ipswich, MA, USA) to create cDNA sequencing libraries. Fragmentation reagent was then added to break the purified mRNA into short fragments at 94 °C for 15 min. First-strand cDNA synthesis was conducted using ProtoScript II reverse transcriptase (NEB, Ipswich, MA, USA) and random primers (NEB). A second strand cDNA was generated using Second Strand Synthesis Enzyme Mix (NEB) and then purified using AMPure XP Beads (Beckman Coulter, Beverly, USA). End repair, dA tailing, and sequencing adapter ligation were performed, followed by cDNA purification with AMPure XP beads (Beckman Coulter) and 12 cycles of polymerase chain reaction (PCR) amplification. Then, the libraries were purified by AMPure XP beads (Beckman Coulter). Subsequently, PCR products with lengths of 350–450 bp were selected in 2% agarose gel and quantified with QUBIT DNA HS Assay Kit (Invitrogen Corporation, Carlsbad, CA). Quality of cDNA library was evaluated by 2100 Bioanalyzer chip (Agilent). Libraries were then denatured using sodium hydroxide (NaOH) and diluted to a final concentration of 20 pM. Finally, clusters for RNA-seq were generated on flow cell using TruSeq Rapid PE Cluster Kit (Illumina). After cluster generation, cDNA libraries were sequenced by 2 × 125 paired-end sequencing on an Illumina HiSeq. 2500 platform (Illumina, San Diego, CA, USA). Raw data were stored in FASTQ format. Complete sequencing data obtained in this study have been submitted to the National Center for Biotechnology Information (NCBI) Sequence Read Archive with accession number SRP110797.

### Sequencing quality control and data analysis

Quality of raw sequences was assessed using FastQC (http://www.bioinformatics.babraham.ac.uk/projects/fastqc/). Adaptor sequences, unknown sequences (N), low-quality reads (Q < 20), and their paired sequences measuring less than 50 bases were eliminated from raw reads using Next Generation Sequencing Quality Control Toolkit version 2.3.3.

Reference genome and gene annotation files were downloaded from Ensembl (http://www.ensembl.org/info/data/ftp/index.html). A set of genomic index files of reference genome was built using Bowtie version 2.2.5, and clean reads were mapped to the *Sus scrofa* reference genome (Sscrofa10.2) from NCBI using Tophat version 2.1.0^45^. Supplementary Table 1 presents the detailed alignment information, including total numbers of reads (37.28–42.34 million) and mapped rate (80.7–85.7%). Then, the transcripts were assembled using Cufflinks version 2.2.1. Transcript files generated by Cufflinks were added to a single-merged transcriptome annotation using Cuffmerge version 2.2.1. DEGs and transcripts were identified between different sample groups using Cuffdiff version 2.2.1^46^. Gene expression values were calculated by counting the number of fragments per kilobase of transcript per million mapped fragments. Cuffdiff applies a corrected p-value, known as q-value, to measure significant differences between two sample groups (*q* < 0.05).

### Quantitative reverse transcription PCR (RT-qPCR) validation for RNA-seq

DEGs were validated by RT-qPCR for the following transcripts: *IFN-γ*, *CXCR4*, *CCR1*, *CCL8*, *CCL4*, *IL1B*, and *CXCL8*. One microgram of total RNA per sample was used to perform first-strand cDNA synthesis using a PrimeScript™ RT (Perfect Real Time) reagent kit with gDNA Eraser (Takara, Tokyo, Japan). RT reaction was performed at 37 °C for 15 min and 85 °C for 5 s. Gene-specific primers were designed according to their gene sequences (Supplementary Table 2). cDNA samples were then quantified with RT-qPCR using LightCycler® 480 Real-Time PCR System (Roche, Hercules, CA, USA). RT-qPCR was carried out in a reaction volume of 20 µL with Roche SYBR Green PCR Kit (Roche, Hercules, CA, USA) as follows: 95 °C for 5 min, followed by 40 cycles of 95 °C for 5 s, 60 °C for 15 s, and 72 °C for 15 s. *GAPDH* gene was selected as internal control to normalize cDNA input. Three technical replicates were conducted on each cDNA, and average Ct value was used for further analysis. Amplification efficiencies were close to 100%. Relative expression values were calculated using the 2^−ΔΔCt^ method.

### Gene ontology (GO) terms and Kyoto Encyclopedia of genes and genome (KEGG) pathways

DEGs with criteria of *q* < 0.05 and |fold change| (|FC|) > 1.5 were selected to annotate biological functions. GO term and KEGG pathway analyses of DEGs were performed on an online bioinformatics system (DAVID) (https://david.ncifcrf.gov/)^47^. False discovery rate (FDR) was determined to correct p-values. Significant GO categories were enriched (*P* < 0.01, FDR < 0.05), whereas only pathway categories with *P* < 0.05 were selected.

### Protein–Protein interaction (PPI) network analysis

DEGs were inputted into STRING v10 to generate a PPI network with a reliability score higher than 0.4. STRING database was used to predict physical/functional PPIs from four sources, namely, text mining, high-throughput experiments, coexpression, and databases^48^.

### Statistical analyses

Student’s *t*-test was conducted to analyze antibody levels (ELISA) and mRNA expression levels (RT-qPCR) between the two groups. A p-value less than 0.05 was considered statistically significant for all analyses.

## Electronic supplementary material

Supplementary figures and tables
