# Supplementary material for: Transcriptome of Porcine PBMCs over Two Generations Reveals Key Genes and Pathways Associated with Variable Antibody Responses post PRRSV Vaccination
Source: Sci Rep. 2018 Feb 6;8:2460. doi: 10.1038/s41598-018-20701-w (PMC5802836; doi:10.1038/s41598-018-20701-w)
Supplement: Supplementary file 1 — Supplementary figures and tables [file 41598_2018_20701_MOESM1_ESM.pdf]

Transcriptome of Porcine PBMCs over Two Generations Reveals Key Genes and Pathways Associated with Variable Antibody Responses post PRRSV Vaccination

Ting Yang[1], Fengxia Zhang[1], Liwei Zhai[1], Weiyong He[2], Zhen Tan[1], Yangyang Sun[1], Yuan Wang[1], Lei Liu[1], Chao Ning[1], Weiliang Zhou[3], Hong Ao[4], Chuduan Wang[1*] and Ying Yu[1*]

[1]Key Laboratory of Animal Genetics, Breeding, and Reproduction, Ministry of Agriculture & National Engineering Laboratory for Animal Breeding, College of Animal Sciences and Technology, China Agricultural University, Beijing, 100193, China. [2]State Key Laboratory of Agrobiotechnology, Department of Microbiology and Immunology, College of Biological Science, China Agricultural University, Beijing, 100193, China. [3]Tianjin Ninghe primary pig breeding farm, Ninghe, 301500, Tianjin, China. [4]State Key Laboratory for Animal Nutrition, Key Laboratory for Domestic Animal Genetic Resources and Breeding of the Ministry of Agriculture of China, Institute of Animal Science, Chinese Academy of Agricultural Sciences, Beijing, 100193, China

***Correspondence:**
Corresponding authors
*****cdwang@cau.edu.cn (CDW); yuying@cau.edu.cn (YY)

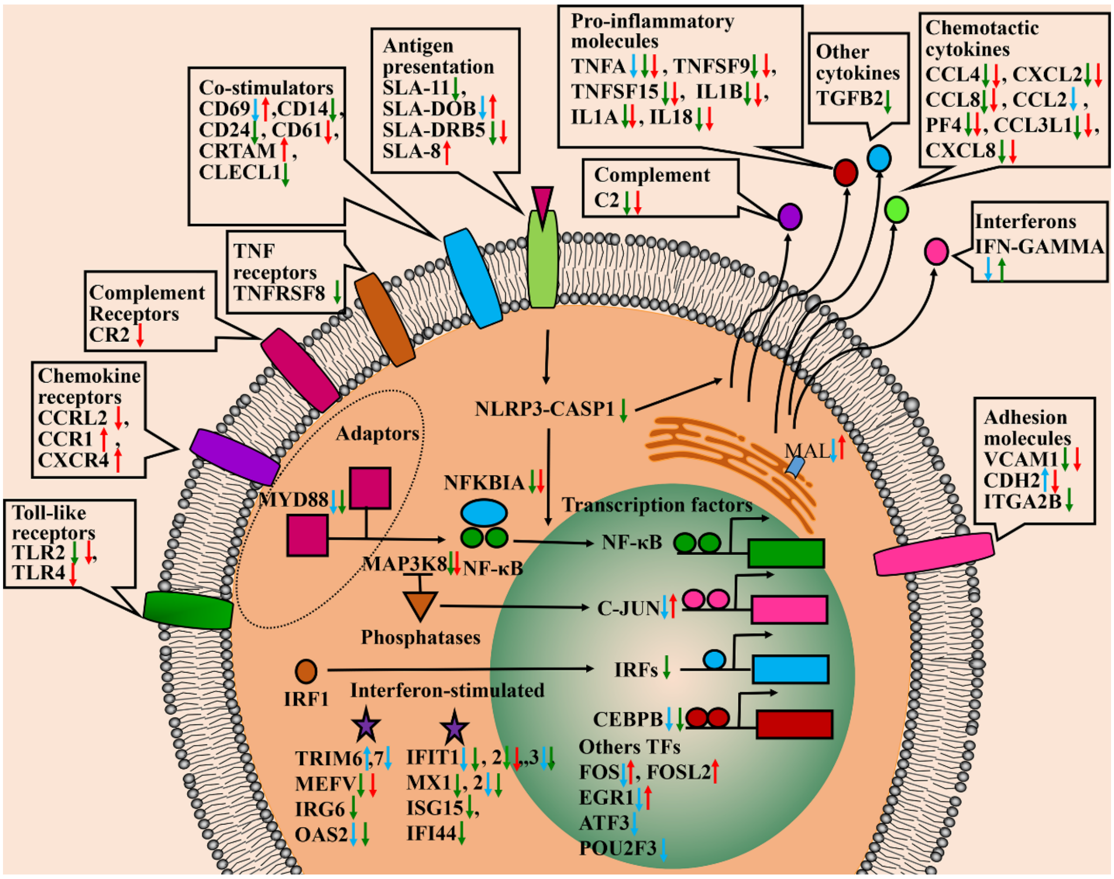

Supplementary Figure S1. Significant immune responses of porcine PBMCs at 35 days after PRRSV-MLV vaccination. DEGs of host response and their functions inside and outside PBMCs. Genes marked with blue, green, and red color arrows represent HA versus MA, HA versus LA, and MA versus LA, respectively. Genes marked with a downward arrow were downregulated, and the others were upregulated.

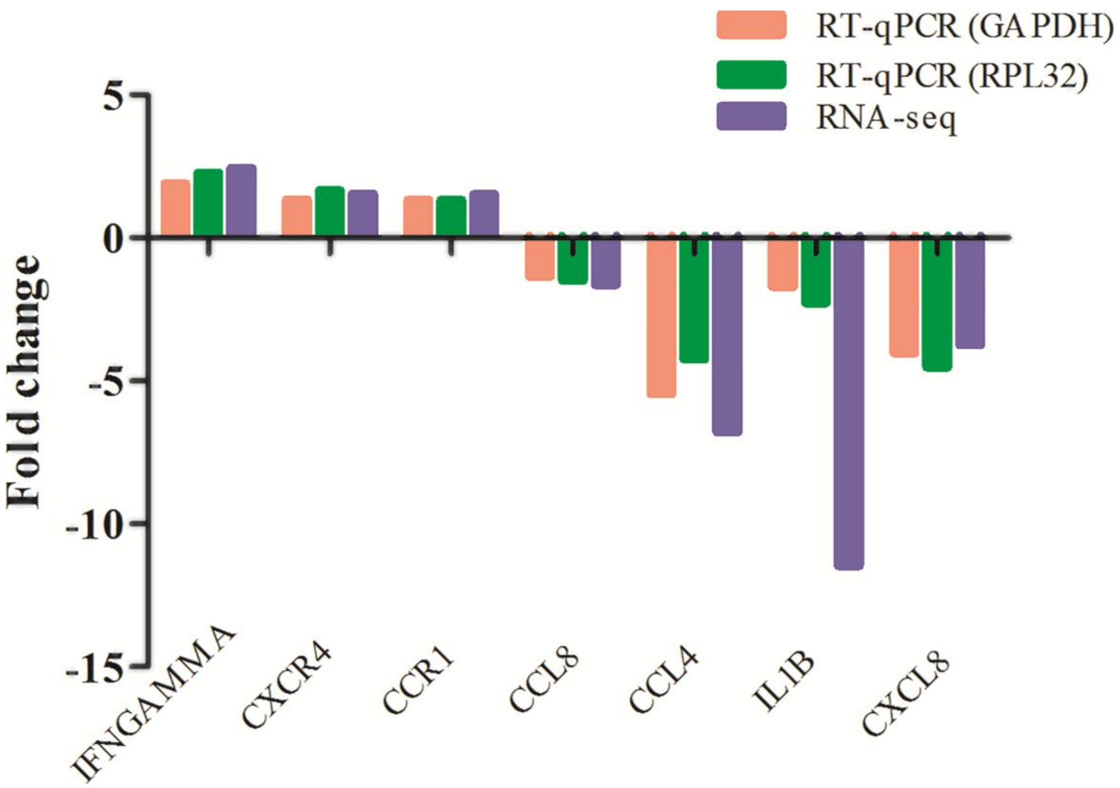

Supplementary Figure S2. RT-qPCR validation of RNA-Seq data. *GAPDH* and *RPL32* genes were as internal controls seperately.

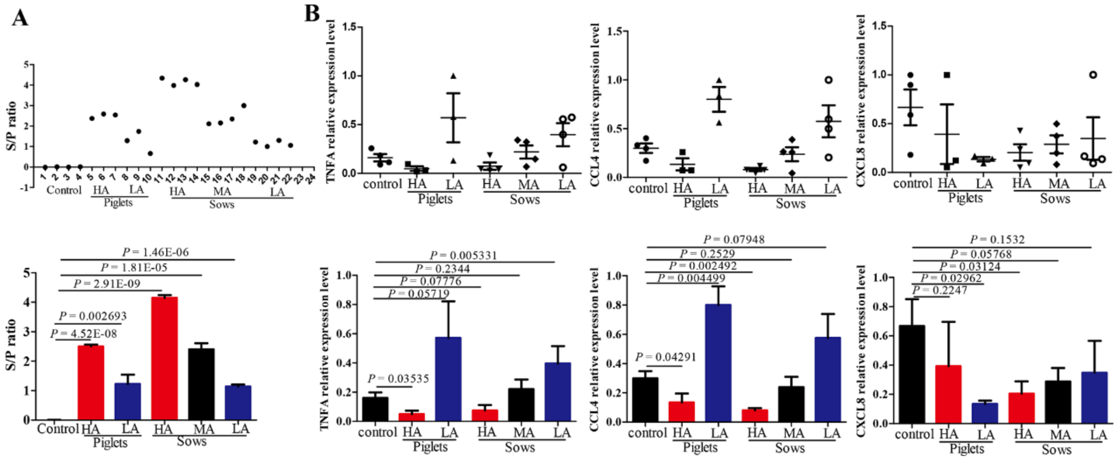

Supplementary Figure S3. Immune responses and gene expression quantification of naive piglets, vaccinated piglets, and pregnant sows to PRRSV-MLV vaccination. (A) S/P ratio among controls (n = 4), vaccinated piglets (n = 3), and sows (n = 4). (B) Expression levels of *TNF-α*, *CCL4*, and *CXCL8* genes in controls (n = 4), vaccinated piglets (n = 3), and sows (n = 4) with RT-qPCR. *P* means significant level of *t*-test.

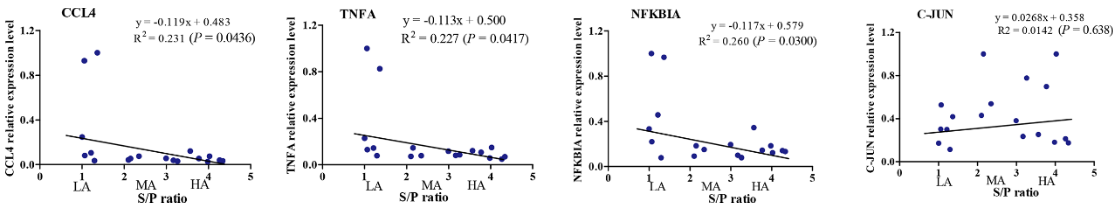

Supplementary Figure S4. Regression analysis of divergent PRRSV-MLV-specific antibody levels (x-axis) with gene expression levels (y-axis) in sows and piglets. HA: high antibody group (n = 8); MA: median antibody group (n = 4); LA: low antibody group (n = 6).

Supplementary Table 1. The basic statistics of sequencing reads aligned to genome for different pigs.

Sample ID	Total tags (Million)	Mapping rating %
H37	37.8	82.1%
H48	41.58	81.5%
H78	42.34	83.9%
H90	37.5	83.2%
M35	39.47	85.7%
M44	40.14	81.9%
M49	38.92	82.6%
M91	42.16	81.2%
L39	41.12	81.8%
L45	40.44	85.1%
L51	37.28	80.7%
L63	38.22	83.4%

Supplementary Table 1. Primer sequences for qRT-PCR validation

Gene name	Primer sequence (5’-3’)	Amplified size (bp)	Annealing temperature (°C)
CCL4	F:CTGCTGCTTCACATACACCGT<br>R:CAGACCTGCCTGCCCTTTT	123	60
CCL8	F: CAAGAATCACCAACAGCCAGT<br>R: CAGTCCAGGTAGGAAGGTTCAA	164	58
CCR1	F:CAGAAACAAAGACTTCGTGGACA<br>R: CACAGGTCAAAGGAAATGGTT	96	58
CXCL8	F: TAGGACCAGAGCCAGGAAGA<br>R: AATTTGGGGTGGAAAGGTGT	191	58
CXCR4	F: CGGGTTCCGTATATTCACTTCA<br>R: ACCCACTATGCCAGTTAAGAAGAT	163	60
IFN-γ	F:CCTAAATGGTAGCTCTGGGAAAC<br>R:ATGAGTTCACTGATGGCTTTGC	99	60
IL1B	F:CTGTTATTTGAGGCTGATGGC<br>R:TCATCGGCTCCTCCTTTGC	166	60
IFNα	F:GGCACAAATGAGGAGAATC<br>R:CACAGCCAGGATGGAGTC	307	60
TLR3	F:AAAATCTCCAAGAGCTTCTATTAGCAA<br>R:TTGTATTTGATTTGATGACAACTCTAATCTTT	119	60
RPL32	F:CGGAAGTTTCTGGTACACAATGTAA<br>R:TGGAAGAGACGTTGTGAGCAA	94	58-60
GAPDH	F:ACTCACTCTTCTACCTTTGATGCT<br>R:TGTTGCTGTAGCCAAATTCA	100	58-60
